# Mechanical complexity of living cells can be mapped onto simple homogeneous equivalents

**DOI:** 10.1007/s10237-024-01823-9

**Published:** 2024-02-27

**Authors:** Sebastian Wohlrab, Sebastian Mueller, Stephan Gekle

**Affiliations:** Theoretische Physik VI, Biofluid Simulation and Modeling, Universität Bayreuth, 95440 Bayreuth, Germany

**Keywords:** Cell mechanics, Atomic force microscopy, Cell elasticity, Shear flow, Cell nucleus

## Abstract

Biological cells are built up from different constituents of varying size and stiffness which all contribute to the cell’s mechanical properties. Despite this heterogeneity, in the analysis of experimental measurements one often assumes a strongly simplified homogeneous cell and thus a single elastic modulus is assigned to the entire cell. This ad-hoc simplification has so far mostly been used without proper justification. Here, we use computer simulations to show that indeed a mechanically heterogeneous cell can effectively be replaced by a homogeneous equivalent cell with a volume averaged elastic modulus. To demonstrate the validity of this approach, we investigate a hyperelastic cell with a heterogeneous interior under compression and in shear/channel flow mimicking atomic force and microfluidic measurements, respectively. We find that the homogeneous equivalent cell reproduces quantitatively the behavior of its heterogeneous counterpart, and that this equality is largely independent of the stiffness or spatial distribution of the heterogeneity.

## Introduction

Cellular mechanics is an intensively investigated topic and a variety of experimental methods has been developed to characterize the elasticity of biological cells (Kollmannsberger and Fry 2011; Wu et al. 2018; Guck 2019). One of the main techniques is atomic force microscopy (Guz et al. 2014; Lulevich et al. 2006; Ladjal et al. 2009; Hecht et al. 2015; Sancho et al. 2017; Müller et al. 2021; Hobson et al. 2020; Abuhattum et al. 2022). Other micromechanical evaluation techniques include the flow through highly confined microchannels (Urbanska et al. 2020; Otto et al. 2015; Fregin et al. 2019; Rowat et al. 2013; Lange et al. 2017; Toepfner et al. 2018; Lange et al. 2015), or mechanical testing in larger channels (Gerum et al. 2022). Both kinds of experiments are most commonly analyzed using a mechanical model which treats the entire cell as one continuous entity endowed with a single elastic modulus and, sometimes, viscosity. This simple cell model has also been used in a series of computer simulations (Rosti et al. 2018; Saadat et al. 2018; Müller et al. 2021; Wittwer et al. 2023; Müller et al. 2023).

At the same time, however, it is known that the different constituents of the cell, e. g., cortex, cytoplasm, and especially the nucleus, all have different mechanical properties (Cordes et al. 2020; Zhelev et al. 1994; Lange et al. 2017; Lykov et al. 2017; Mietke et al. 2015; Caille et al. 2002; Cao et al. 2013; Kolker et al. 2022). Often the size of these constituents, especially that of the stiff nucleus, is of the same order as the size of the cell as a whole. This makes the application of classical homogenization theory difficult as there is no obvious separation of length scales. It is thus tempting to ask why the simplistic assumption of a homogeneous cell appears to work so surprisingly well in many situations.

In this work, we therefore systematically probe the possibility to substitute any heterogeneously constituted cell by a simple homogeneous cell with a single effective elastic Young’s modulus. For that, we first construct a well-defined heterogeneous cell with an inclusion of variable stiffness, size, and position. This inclusion mimicks the mechanical influence of the cell nucleus which due to its different physiological composition is mechanically stiffer than the surrounding soft cytoskeleton. As a second model system, we consider a heterogeneous cell with a spatially random stiffness distribution. From the volume averaged mean of the constituents’ Young’s moduli we define an effective Young’s modulus corresponding to a homogeneous equivalent cell. With these at hand, we perform AFM compression simulations as well as microfluidic shear flow and channel flow computations. We find excellent agreement of the resulting force versus deformation curves in compression and strain versus fluid forces in shear and mostly also in channel flow. Only at high flow rates in channel flow small deviations between the heterogeneous cell and its homogeneous equivalent start to appear. Through variation of stiffness, size, position, and shape, of the inhomogeneity we show that neither of these factors have a significant impact on the cell’s mechanical behavior. Any kind of intracellular mechanical diversity can hence be effectively described using our proposed homogeneous equivalent cell.

## Methods and setup

### Cell models

#### Heterogeneous cell with nucleus

**Fig. 1:**
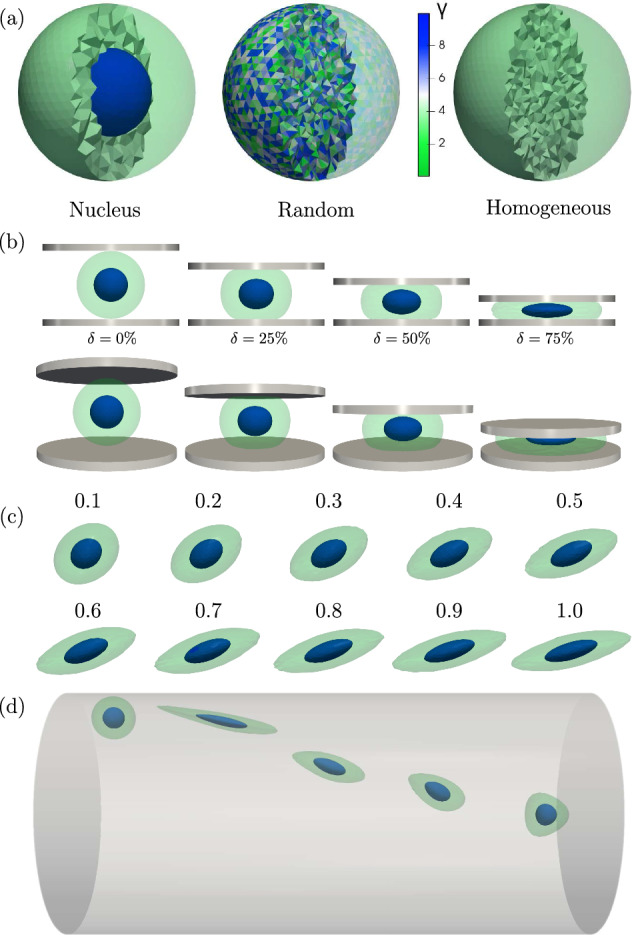
**a** Illustrations of our heterogeneous cells and their homogeneous equivalent showing the stiffness ratio $\gamma$ of the individual tetrahedra. **b** Illustration of an example heterogeneous cell under compression at different values of the deformation $\delta$. **c** The stationary cell shape of a cell with a centered inhomogeneity in linear shear flow with increasing capillary number $\textrm{Ca}$ (number in image corresponds to $\text {Ca}\cdot \gamma _\text {eff}$, see Fig. 5). **d** Our heterogeneous cell flowing through a cylindrical capillary migrates towards the symmetry axes while maintaining an ellipsoidal shape. At the center, it assumes a bullet-like shape

Our first model is a heterogeneous elastic cell with a stiff nucleus surrounded by a softer cytoskeleton. We model this nucleated cell as a sphere of radius $R_\text {c}$ which contains a spherical inclusion of radius $R_\textrm{n}$ inside the cell volume, as shown in Fig. 1a. This model will be labeled “Nucleus” in the plots. We discretize both volumes using tetrahedrons and apply the neo-Hookean strain energy computations from (Müller et al. 2021) in both parts. We checked that similar results are obtained when using the Mooney–Rivlin strain energy as detailed in Appendix. Properties differentiating between the nucleus and the cytoskeleton are distinguished by the subscripts “$\textrm{n}$” and “$\textrm{c}$”, respectively. To parametrize the stiffness we choose the Young’s moduli $E_\textrm{n}$ and $E_\textrm{c}$ of the nucleus and the cytoskeleton, respectively. The Poisson’s ratio is $\nu =0.48$ in all simulations, which ensures sufficient incompressibility while maintaining numerical stability. We note that in this work we only consider the elastic (and partly viscoelastic, see Sect. 2.2.2) response of the cell. Plastic deformation, which requires an active rearrangement of the cytoskeleton, is not considered in this work.

For our systematic analysis, we define the stiffness ratio and the size ratio

1 $$\begin{aligned} \gamma =\frac{E_\textrm{n}}{E_\textrm{c}} \quad \textrm{and} \quad \lambda = \frac{R_\textrm{n}}{R_\text {c}} \,, \end{aligned}$$

with $\gamma >1$ describing a nucleus stiffer than the rest of the cell and $0<\lambda <1$. In some simulations, we position the nucleus offset from the cell’s geometrical center by $d$ in units of the cell radius. Through variation of the control parameters $\gamma$, $\lambda$, and $d$, any kind of spherical inclusion into the cell volume is covered. We discuss the effect of an ellipsoidal inhomogeneity in the last paragraph of Sect. 3.1.

As a reference configuration, from which variations of the control parameters start, we choose $\gamma =2$, $\lambda =\frac{1}{2}$, and $d=0$.

#### Random heterogeneous cell model

As our second model system of a heterogeneous cell, we randomly assign a stiffness ratio $\gamma _i \in [1,10]$ to every of the $N_\textrm{tet}$ individual tetrahedra of the mesh, as shown in Fig. 1a. These cells will be labeled “Random” in plots.

#### Homogeneous equivalent cell model

The purpose of our work is to show that both heterogeneous cell models introduced above show identical mechanical behavior as an equivalent homogeneous cell with properly assigned effective elastic moduli. An illustration is shown in Fig. 1a and will be labeled “Homogeneous” in upcoming plots. Discretization and force computation in the homogeneous cell model proceed in the same way as for the heterogeneous models and as in earlier works (Müller et al. 2021).

To connect a nucleated heterogeneous cell with its homogeneous equivalent, we compute the effective Young’s modulus of the homogeneous equivalent as

2 $$\begin{aligned} E_\textrm{eff}= \frac{1}{V}\left( V_\textrm{c}E_\textrm{c}+ V_\textrm{n}E_\textrm{n}\right) = \bigg [ 1 + (\gamma -1) \lambda ^3 \bigg ] E_\textrm{c}. \end{aligned}$$

Analogously for our random heterogeneous cell model, the effective Young’s modulus is computed as

3 $$\begin{aligned} E_\textrm{eff}= \frac{1}{V} \sum \limits _{i=1}^{N_\textrm{tet}} V_i E_\textrm{c}\gamma _i = E_\textrm{c}\gamma _\textrm{eff}\,, \end{aligned}$$

from the volumes $V_i$ and the Young’s moduli $E_i = \gamma _i E_\textrm{c}$ of the $N_\textrm{tet}$ individual tetrahedra. We note that these definitions correspond to the so-called Voigt modulus in composite material theory (Voigt 1889). For the random heterogeneous cell, we choose the volume averaged stiffness ratio as $\gamma _\textrm{eff}\approx 5.5$.

### Simulation setups

#### Cell simulations under compression

To mimic AFM experiments, we compress our model cells between an upper and a lower plate using the algorithm described in (Müller et al. 2021). Among others, this setup closely approximates the experimental situation of colloidal probe measurements where a large sphere is used to indent the cell, see e.g. (Lulevich et al. 2006). From these quasistatic simulations we obtain the normal force $F$ exerted by the upper plate onto the cell as well as the resulting cell deformation as shown in Fig. 1b. Using the plate-plate distance *D*, we define the dimensionless compression ratio as $\delta = 1 - \frac{ D }{ 2R_\text {c} }$. We perform our simulations up to very large deformations of $\delta ={75}\,\%$ for parameters $\gamma \in {1,2,10,20}$ and $\lambda \in {0.1,0.2,\dots ,0.9}$, as well as for our random heterogeneous cell. We then perform another set of simulations with our homogeneous equivalent cell with the effective Young’s modulus from (2) and (3).

#### Cell simulations in shear flow

As a first flow scenario we use a linear shear flow, where our initially spherical cell deforms into an ellipsoidal body that undergoes a tank-treading motion. To do so, we couple our hyperelastic tetrahedralized mesh to a Lattice Boltzmann flow simulation (Krüger et al. 2017) via an immersed-boundary algorithm using the same procedure as in (Müller et al. 2021, 2023). For the simulations we use the ESPResSo package (Roehm and Arnold 2012; Limbach et al. 2006; ICP-Stuttgart 2006). For simplicity, the interior of the cell is chosen to have the same viscosity as the outer fluid.

Since the cell assumes an ellipsoidal shape, we choose the Taylor deformation parameter (Müller et al. 2021; Saadat et al. 2018)

4 $$\begin{aligned} D= \frac{a-b}{a+b} \end{aligned}$$

with the ellipsoid’s major and minor semi-axis *a* and *b*, respectively, as our measure for the cell deformation. In analogy to the normal force introduced in section 2.2.1, the strength of the shear flow is best characterized using the dimensionless shear rate, or capillary number

5 $$\begin{aligned} \textrm{Ca}=\frac{\eta \kappa }{\mu _\text {eff}} = 2(1+\nu )\frac{\eta \kappa }{E_\text {eff}}\,, \end{aligned}$$

where $\eta$ denotes the surrounding fluid’s dynamic viscosity and $\kappa = \frac{{\partial }u_x}{{\partial }y}$ the constant velocity gradient. Commonly, the shear modulus $\mu$ is used as stiffness parameter for this definition. It relates to the Young’s modulus of the previous section via the Poisson’s as $E=2(1+\nu )\mu$. Hence, the stiffness ratios $\gamma$ and $\gamma _\textrm{eff}$ have the identical value when defined analogously to (1) and (3) via the shear moduli of the nucleus and the cytoskeleton, respectively $\mu _\textrm{n}$ and $\mu _\textrm{c}$.

In Fig. 1c, we show the stationary shape of our heterogeneous cell at various $\textrm{Ca}$. In addition to the ellipsoidal deformation of the entire cell, we find that the centered nucleus, too, deforms into an ellipsoidal manner. However, its deformation is visibly less pronounced. We perform our simulations for $\gamma \in {1,2,10,20}$ and $d\in {0,0.45}$, and with our random heterogeneous cell. Using the effective shear modulus $\mu _\textrm{eff}$ in (5), we compare the heterogeneous cells’ behavior with the master curve describing the homogeneous equivalent cell.

#### Cell simulations in channel flow

In our second flow scenario, we place the initially spherical cell inside a cylindrical channel with radius $R_\textrm{ch}= {50}\,\upmu \hbox {m}$, where an axial pressure gradient *G* drives the Poiseuille flow (Müller et al. 2020). Here, we need to distinguish two important cases as illustrated in Fig. 1d: (i) When placed off-centered the cell will assume an approximately ellipsoidal shape according to the local shear rate. Recently, it has been shown experimentally (Gerum et al. 2022) and numerically (Müller et al. 2023), that a local shear flow approximation is valid for microfluidic and pipe flow applications, given that cells flow off-centered. Due to the fluid’s shear stress, however, the cell continuously migrates from its starting point towards the center where the local shear flow approximation becomes insufficient.

(ii) At the channel center the cell assumes a bullet-like shape due to the symmetrical flow conditions as shown in Fig. 1d. We perform our simulations for $\gamma \in {2,10,20}$ and compare the shape to these to their homogeneous equivalent cell. We also calculate the stress each cell experiences in the center of the channel by averaging over the von Mises stress of all tetrahedra weighted with their undeformed volume. The von-Mises stress is an invariant of the Cauchy stress tensor quantifying multiaxial loading using a single scalar value. It is calculated like so:

6 $$\begin{aligned} \sigma _\text {vM} = \sqrt{\frac{1}{2}\left[ (\sigma _1 - \sigma _2)^2 + (\sigma _2-\sigma _3)^2 + (\sigma _3-\sigma _1)^2\right] } \end{aligned}$$

Where $\sigma _i$ are the eigenvalues of the Cauchy stress tensor, the so-called principal stresses.

## Results

### Cells under compression

**Fig. 2:**
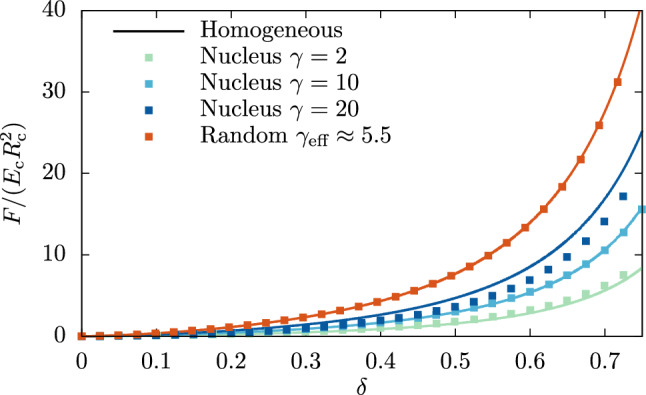
The force versus deformation curve of heterogeneous nucleated cells with constant nuclear size ratio $\lambda =\tfrac{1}{2}$ and varying stiffness ratio $\gamma$ (dots). Increasing the stiffness of the nucleus increases the overall force necessary to compress the cell. Lines show the homogeneous equivalent cell for each $\gamma$ which are in excellent agreement for $\gamma =2, 10$ and still in reasonable agreement for $\gamma =20$

We start with the nucleated cell model from Sect. 2.1.1 and perform compression simulations as described in Sect. 2.2.1 to mimic the situation of an AFM experiment. The ratio between nucleus and cell size is $\lambda =\tfrac{1}{2}$ and the nucleus is placed at the center of the cell. When increasing the stiffness ratio $\gamma$ at a constant size of the nucleus under compression, we find that—as expected—the force needed to compress the cell to a certain deformation $\delta$ increases. This can be seen in Fig. 2, where we plot the dimensionless force $F/(E_\textrm{c}R_\text {c}^2)$ versus the deformation $\delta$. We note that the normalization factor contains the Young’s modulus of the cytoskeleton $E_\textrm{c}$ and therefore is identical for all simulations even with different $\gamma$. We achieve the variation of the stiffness ratio via adjustment of the Young’s modulus of the nucleus while the Young’s modulus of the cytoskeleton remains untouched. The results however are unchanged if instead the Young’s modulus of the cytoskeleton is varied (see Appendix B). In the same manner, we plot in Fig. 2 the data obtained from the simulations performed with the corresponding homogeneous equivalent cells as lines. We find that for almost the entire range of nuclear stiffnesses the deviation from the homogeneous equivalent cell is insignificant. A certain deviation can only be seen for the largest stiffness ratio $\gamma = 20$ which however is most likely beyond the range exhibited by typical cells. Interestingly, our data for $\gamma =10$ matches perfectly with its homogeneous equivalent, whereas the differences for other values of $\gamma$ deviate in different directions. While for $1<\gamma <10$, the heterogeneous cell exhibits slightly stronger strain hardening than the homogeneous equivalent cell, for $\gamma >10$ the strain hardening is instead slightly decreased. We include in the figure the random heterogeneous cell from Sect. 2.1.2 which also shows excellent agreement with its homogeneous equivalent.

**Fig. 3:**
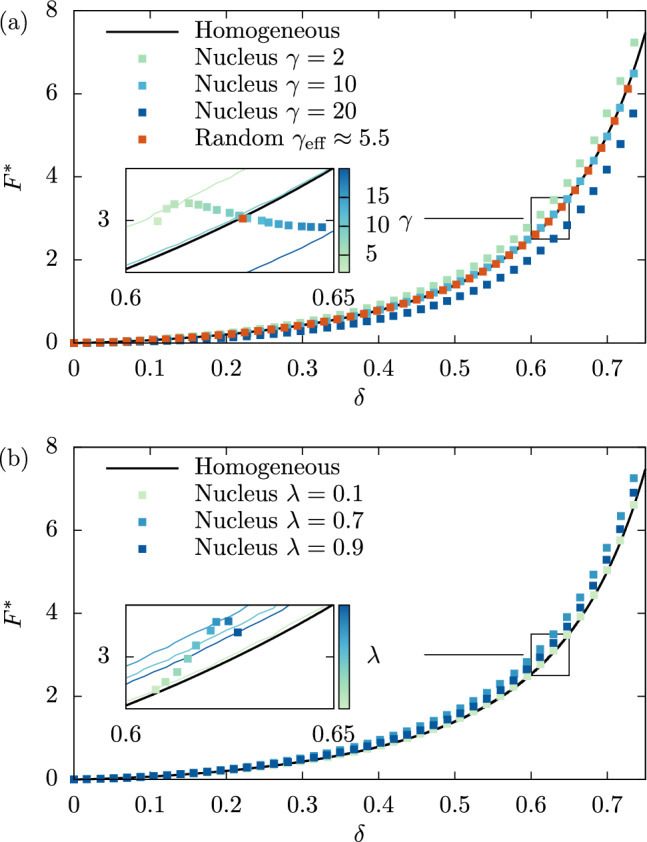
**a** The quality of the homogeneous equivalent when varying the stiffness ratio $\gamma$ can be visualized using the normalized force $F^*$ (7). **b** Variation of the volume ratio $\lambda$ (1) instead of $\gamma$ shows similarly good agreement

To demonstrate the agreement in a more quantitative way, we non-dimensionalize the force using the effective Young’s modulus (2)

7 $$\begin{aligned} F^* = \frac{F}{E_\textrm{eff}R_\text {c}^2} \,, \end{aligned}$$

which is shown in Fig. 3a. Due to this non-dimensionalization, all data for homogeneous cells collapse onto a single master curve. The data of the heterogeneous cells only slightly deviate from this master curve in different directions, which allows us to assess clearly the quality of the homogeneous equivalent description. This is further visualized in the inset of Fig. 3a, where additional values of $\gamma$ are added as well.

Having demonstrated the validity of the homogeneous equivalent for $\lambda =\tfrac{1}{2}$, we now proceed to vary the size of the nucleus at constant stiffness $\gamma =2$. The two limiting cases are $\lambda =0$ (all cytoskeleton) and $\lambda =1$ (all nucleus). In Fig. 3b, the resulting normalized force (7) versus deformation curves for $\lambda =0.1$, $\lambda =0.7$ and $\lambda =0.9$ all show very good agreement with their homogeneous equivalent. Again, we use an inset into Fig. 3b to assess more precisely even small deviations. We note that the deviation attains a maximum around $\lambda \approx 0.7$ which is close to $\lambda =2^{-\frac{1}{3}}\approx 0.79$ as would be obtained for equal volumes of cytoskeleton and nucleus.

**Fig. 4:**
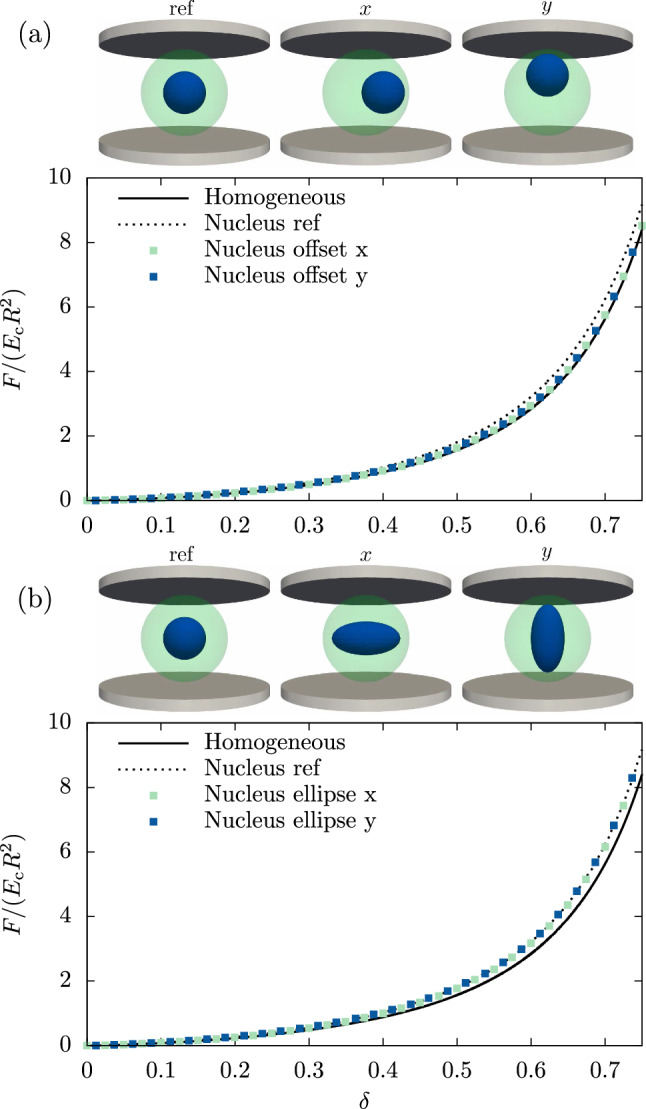
**a** Variation of the position of the nucleus along two independent axes does not affect the accuracy of the homogeneous equivalent. **b** A nucleus with ellipsoidal shape (but same volume) does not notably affect the force versus deformation behavior independent of its orientation

Next, we move the inhomogeneity ($\gamma =2$ and $\lambda =\tfrac{1}{2}$) away from the center to a positions very close to the cell surface, i. e., $d=0.45$. As illustrated in Fig. 4a, we denote with *x* the direction parallel to the plates and with *y* the perpendicular direction. The force versus deformation curves in Fig. 4a show almost perfect overlap, thus demonstrating–somewhat surprisingly–that the position of the nucleus has no influence on the overall mechanical behavior of the cell in AFM compression experiments.

Finally, we alter the shape of the nucleus and replace the centered spherical nucleus with an ellipsoid of equal volume with semi-axes $a\approx 0.8\,R_\text {c}$, $b=c\approx 0.4\,R_\text {c}$. We choose again the parallel (*x*) and perpendicular (*y*) alignment of the major semi-axis, which we compare to the centered spherical nucleus ($\lambda =\tfrac{1}{2}$) denoted by ”ref”, as shown in Fig. 4b. The resulting force versus deformation curves for ($\gamma =2$) in Fig. 4b underline that a variation of the nucleus shape effectively does not affect the compression behavior of a cell. Due to imperfections in the computational mesh, cells with their ellipsoidal nucleus aligned along the *y*-axis tend to topple during deformation when using large stiffness ratios (broken rotational symmetry, non-zero torque). We therefore discuss here only the $\gamma =2$ case, where no toppling is present and the results of both geometries are accurate.

From the data in this section, we conclude that in compression scenarios a heterogeneous cell can in practice be replaced with a homogeneous equivalent cell with a volume averaged Young’s modulus, since neither the stiffness difference nor the size, the position, or the shape, of the inhomogeneity have a significant impact on the force necessary to produce a certain cell deformation. Furthermore, our results might open a novel route for estimating nuclear stiffness: treating the cell by a drug which affects cytoskeletal—but not nuclear–stiffness in a known way and measuring a series of cell stiffnesses would allow one to infer nuclear stiffness.

### Cells in linear shear flow

Besides AFM measurements of cell deformation in shear flow has recently been introduced as an efficient characterization technique for cell mechanics (Gerum et al. 2022). We proceed to demonstrate the possibility of replacing heterogeneous cells with homogeneous cells also in this scenario. We start by putting an initially spherical heterogeneous cell ($\gamma =2$, $\lambda =\tfrac{1}{2}$, and $d=0$) in a linear shear flow with shear rate $\kappa$. After a transient time span the cell shape becomes stationary (cf. Fig. 1c) and the cell undergoes a continuous tank-treading motion. To quantify the amount of cell deformation we use the Taylor deformation parameter $D$ introduced in (4). To quantify the strength of the applied shear force, we use the Capillary number defined in (5).

**Fig. 5:**
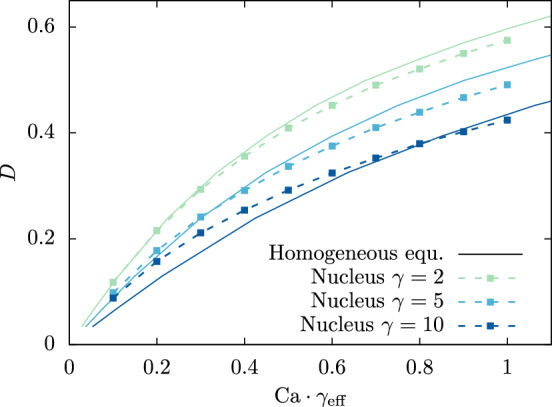
Taylor deformation of the nucleated heterogeneous cell for different capillary number in shear flow. The solid lines represent the curves of their respective homogeneous equivalent cells

In Fig. 5 we find excellent agreement between the heterogeneous nucleated cell and its homogeneous equivalent, when plotting the stationary value of $D$ obtained from the simulation with $\gamma =2$ as a function of $\textrm{Ca}$. In accordance with the compression simulations of Fig. 2a, we find that the heterogeneous cell with nucleus at a stiffness ratio $\gamma =2$ yields a slightly lower deformation than its homogeneous equivalent. Figure 5 furthermore contains results for $\gamma =5$ and 10 which are generally also in very good agreement with their homogeneous equivalent over a wide range of deformations. Analogous to Sect. 3.1 we achieve the variation of $\gamma$ by only tuning the shear modulus of the nucleus. The same results can be achieved via altering the cytoskeleton instead (see Appendix B). Unlike in our AFM simulations the degree of agreement between the inhomogeneous cell and its homogeneous counterpart depends visibly on the amount of strain the cells experience.

**Fig. 6:**
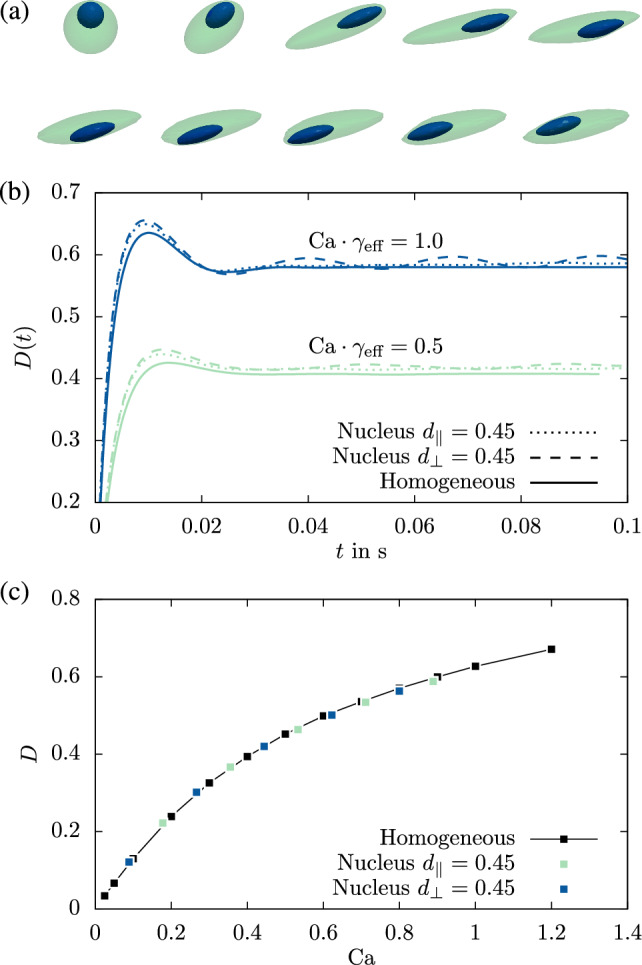
**a** Time series snapshots for the heterogeneous cell with nucleus displaced within the shear plane. Due to the rotation of the cellular material, the bump produced by the inhomogeneity travels around the cell. **b** Time development of the Taylor deformation $D(t)$ for a heterogeneous cell with the nucleus displaced either within or perpendicular to the shear plane when compared to the time development of a homogeneous cell. The case with the nucleus displaced within the shear plane shows small periodic oscillations. **c** Average $D$ of the cell with off-centered nucleus compared to the data of Fig. 5a shows the validity of the homogeneous equivalent also for this situation

We next investigate the behavior of the cell when the nucleus is positioned away from the cell center. A time series of snapshots for a nucleus displacement $d_\parallel =0.45$ within the shear plane is shown in Fig. 6a. These snapshots depict the tank-treading motion of the entire cell, where the nucleus produces a bump at the cell surface. This behavior is clearly reflected in the temporal dynamics of the Taylor deformation (4) shown in Fig. 6b, where $D$ is plotted as function of time *t*. Interestingly, if instead the nucleus is displaced perpendicular to the shear plane at $d_\perp =0.45$, the same stationary behavior as for a centered nucleus is obtained in Fig. 6b. The time average of the deformation in this state is shown in Fig. 6c for both nucleus offsets as well as the corresponding homogeneous equivalent cell. It becomes clear that, despite the periodic oscillations for the nucleus displaced in-plane, the time-averaged deformation of cells with off-centered nuclei perfectly matches the homogeneous equivalent.

### Cell in capillary flow

The final scenario which we consider in this work is a cell flowing through a microchannel as occurring, e.g., in RT-DC characterization experiments (Otto et al. 2015; Fregin et al. 2019). The two major differences between the pressure driven flow through a microchannel and the simple shear flow scenario of the previous section are (i) the nonlinearity of the velocity profile and (ii)  the symmetry conditions at the channel axis.

**Fig. 7:**
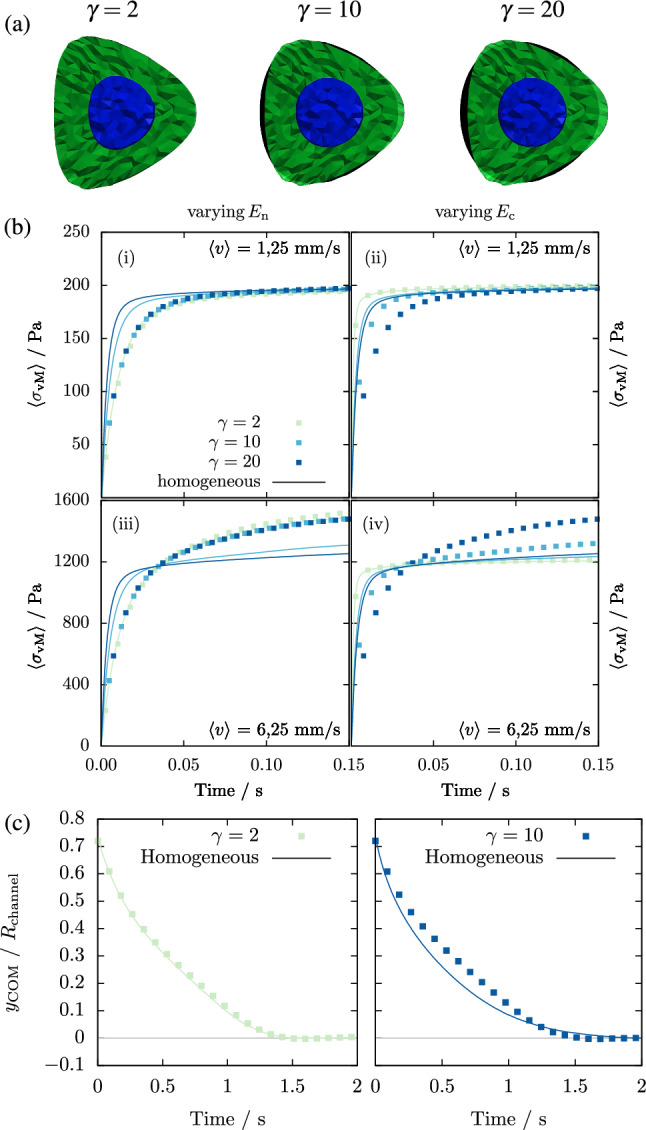
**a** Snapshots of the heterogeneous cell with nucleus and its homogeneous equivalent when flowing at the center of the channel for $\gamma =2$, 10 and 20. The black area shows a slice of the homogeneous equivalent cell while the green/blue overlay depicts the heterogeneous cell with nucleus. **b** Time evolution of the average von-Mises stress inside heterogeneous cells at two different fluid velocities. The shell Young’s modulus is kept fixed at $E_\text {c} = {1}\,kPa$ in (i) and (iii) while in (ii) and (iv) the nucleus Young’s modulus is fixed at 20 kPa. At high flow velocity the volume averaged Young’s modulus model only yields accurate results for small $\gamma$. Generally the stress inside the cell is dominated by the cytoskeleton stiffness. **c** A slight variation of the dynamic of the migration inside the channel occurs for large $\gamma$. However the time needed for the cell to reach the center remains similar

When placing a homogeneous cell at the center of the channel at an average flow velocity of 5 mm/s, it assumes a stationary bullet-like shape as shown in previous works, e.g. (Müller et al. 2023). Figure 7a shows that this behavior is qualitatively retained for heterogeneous nucleated cells with stiffness ratios $\gamma =2$, 10 and 20. The figure furthermore demonstrates reasonably good agreement with the shape of the homogeneous equivalent cell in the same flow. For higher $\gamma$ this is again somewhat surprising as the cytoskeleton in contact with the surrounding fluid is significantly softer for the heterogeneous cell (2.125 times softer at $\gamma =10$).

As a measure for the cell stress, we depict in Fig. 7b the time evolution of the average von Mises stress of our heterogeneous cells and their homogeneous equivalent at low and high flow velocity. As the cell stress needs to counterbalance the fluid stress, it is not surprising that at low channel velocity (Fig. 7b-i, ii) the cell stress tends to a value independent of the cell geometry and stiffness. In addition, we find very good agreement between the heterogeneous and the homogeneous equivalent when the stiffness ratio $\gamma$ is tuned by varying the nucleus stiffness (Fig. 7b-i). If, however, we vary the cytoskeleton stiffness as shown in Fig. 7b-ii, we find a small, but notable deviation for higher values of $\gamma$ for this system (this was not the case in the other systems considered so far as shown in Appendix B). This deviation can be explained by the fact that the nucleus is located on the symmetry axis of the channel where the fluid stress vanishes  (Müller et al. 2020), while it becomes larger towards the edges of the cell and thus primarily acts on the cytoskeleton part of the cell. Therefore we can assume the influence of the nucleus to be substantially weaker here as compared to off-center flow, where the whole cell surface is subjected to large fluid stresses. At high fluid velocity (Fig. 7b-ii, iv) the deviation between heterogeneous cell and homogeneous equivalent becomes somewhat more pronounced, but still does not exceed 20%.

When the cell is placed off-center the gradient in shear rate causes it to migrate towards the center (See Fig. 1d). The speed of migration for both heterogeneous and homogeneous cells is shown in Fig. 7c. For $\gamma =2$ the migration paths are identical. For higher $\gamma$ slight differences in the migration paths become visible, however the duration until the middle of the channel is reached remains very similar.

Because differences between the homogeneous and heterogeneous setup only manifest for the average cell stress in case of a large stiffness difference between nucleus and cytoskeleton and a cell flowing exactly centered through the channel, we conclude that our proposed homogeneous equivalent description is still overall valid in capillary flow.

## Conclusion

In this work, we presented a systematic numerical demonstration that the mechanics of highly heterogeneous cells can be replaced by a simple homogeneous equivalent with a single effective elastic modulus. The effective modulus is obtained by simply taking the volume average over the different moduli characterizing the heterogeneous cell. We demonstrated this equivalence for three scenarios each corresponding to a commonly used experimental measurement technique: (i) AFM compression, (ii) a cell in shear flow and (iii) a cell flowing in a microchannel. The approach is valid for nucleated cells with different cytoskeleton/nucleus stiffness ratios, cytoskeleton/nucleus size ratios, nucleus positions and even nucleus shapes. This underlines the robustness of our findings.

Our results thus validate in hindsight the simplifying approaches taken in many previous experimental and computational works, but also provide a solid basis on which future experimental data can be analyzed and physically reliable computer simulations can be constructed.

## Figures and Tables

**Fig. 8:**
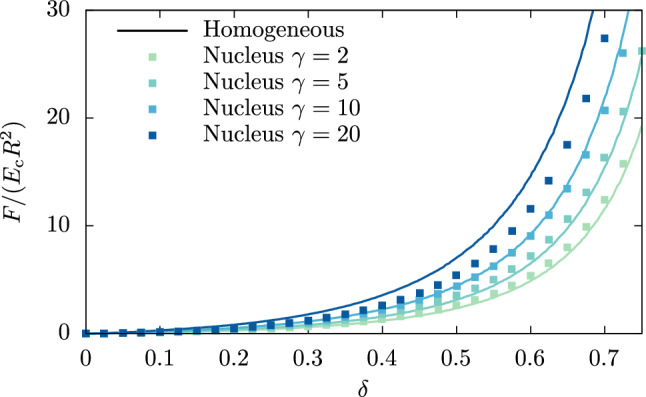
The force versus deformation behavior of heterogeneous cells using the Mooney–Rivlin model. The cell parts material constants are set fractions of the given shear modulus: $\mu _1 = 0.25\mu , \mu _2 = 0.75\mu$. The homogeneous equivalent cell approximation appears as good as when using the Neo-Hookean model (Fig. 2 of the manuscript)

**Fig. 9:**
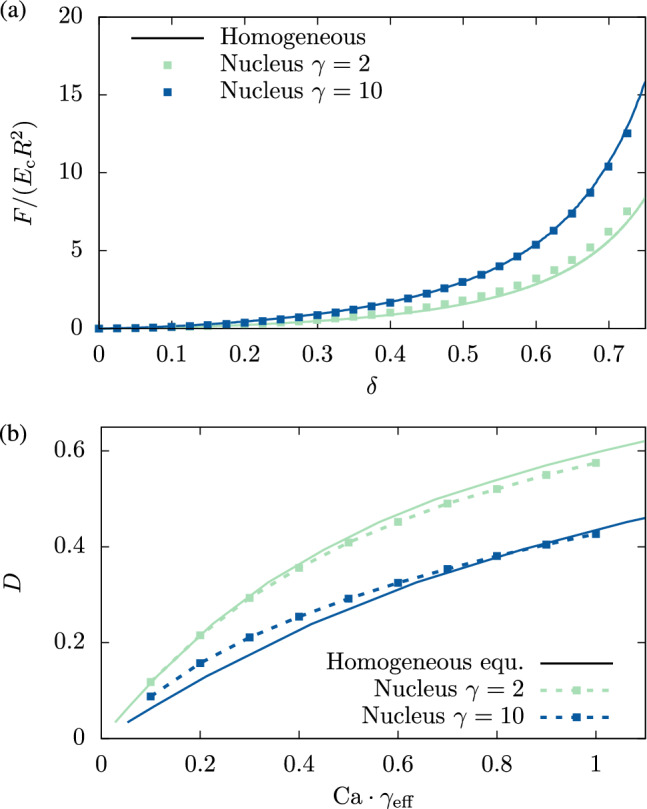
Reproducing Fig. 2a and Fig. 5b, but this time via varying the mechanical properties of the cytoskeleton. The results remain the same

## Appendix A Mooney–Rivlin strain energy computations

As described in Sect. 2.1.1 we use Neo-Hookean strain energy computations for the cells dynamics. The more sophisticated Mooney–Rivlin model in (Müller et al. 2021) uses two separate shear moduli $\mu _1$ and $\mu _2$ for computing the strain energy density of a tetrahedron:

A1 $$\begin{aligned} U_\text {MR} = \frac{\mu _1}{2}(I-3)+\frac{\mu _2}{2}(K-3)+\frac{\kappa }{2}(J-1)^2 \end{aligned}$$

Here *I*, *J* and *K* are invariants of the deformation tensor and $\mu _1$,$\mu _2$ and $\kappa$ relate to Young’s modulus and Poisson ratio like so:

A2 $$\begin{aligned} \mu _1 + \mu _2 = \mu = \frac{E}{2(1+\nu )} \qquad \qquad \kappa = \frac{E}{3(1-2\nu )} \end{aligned}$$

We tested whether the effective Youngs modulus model for a homogeneous equivalent cell still works when using the Mooney–Rivlin model by deriving the equivalent shear modulus of both the nucleus and cytoskeleton: $\mu = \mu _1 + \mu _2$. Figure 8 shows similarly good agreements compared to above between heterogeneous and homogeneous setups which further supports the validity of our model.

## Appendix B Alternative method: varying Young’s/shear modulus of the cytoskeleton

So far we varied primarily the mechanical parameters of the nucleus to study the effect of heterogeneity. Our results however are independent on whether the nucleus or the cytoskeleton is varied. In Fig. 9 previous figures are reproduced, this time via changing the Young’s/shear modulus of the cytoskeleton. As one can see, the results are indistinguishable from Figs. 2 and 5 respectively.
